# Barriers and facilitators to accessing and utilising post-treatment psychosocial support by Black men treated for prostate cancer—a systematic review and qualitative synthesis

**DOI:** 10.1007/s00520-021-06716-6

**Published:** 2022-01-04

**Authors:** Olufikayo O. Bamidele, Obrey Alexis, Motolani Ogunsanya, Sarah Greenley, Aaron Worsley, Elizabeth D. Mitchell

**Affiliations:** 1 Institute for Clinical and Applied Health Research, Hull York Medical School, University of Hull, Hull, HU6 7RX UK; 2 Faculty of Health and Life Sciences, Oxford Brookes University, Joel Joffe Building, Delta 900, Welton Way, Swindon, SN5 7XQ UK; 3 Department of Pharmacy, Clinical & Administrative Sciences, College of Pharmacy, The University of Oklahoma Health Sciences Center, Oklahoma City, OK USA; 4 Cancer Research Group, Hull York Medical School, University of Hull, Hull, HU6 7RX UK; 5 Directorate of Learning Resources, Oxford Brookes University, Oxford, OX3 OBP UK; 6 Hull York Medical School, University of Hull, Hull, HU6 7RX UK

**Keywords:** Prostate cancer, Black men, Psychosocial support, Barriers, Facilitators, Systematic review

## Abstract

**Purpose:**

To synthesise findings from published studies on barriers and facilitators to Black men accessing and utilising post-treatment psychosocial support after prostate cancer (CaP) treatment.

**Methods:**

Searches of Medline, Embase, PsycInfo, Cochrane Database of Systematic Reviews and Central, CINAHL plus and Scopus were undertaken from inception to May 2021. English language studies involving Black men aged ≥18 and reporting experiences of, or suggestions for, psychosocial support after CaP treatment were included. Low or moderate quality studies were excluded. Searches identified 4,453 articles and following deduplication, 2,325 were screened for eligibility. Two independent reviewers carried out screening, quality appraisal and data extraction. Data were analysed using thematic synthesis.

**Results:**

Ten qualitative studies involving 139 Black men were included. Data analysis identified four analytical constructs: experience of psychosocial support for dealing with treatment side effects (including impact on self-esteem and fear of recurrence); barriers to use of psychosocial support (such as perceptions of masculinity and stigma around sexual dysfunction); facilitators to use of psychosocial support (including the influence of others and self-motivation); and practical solutions for designing and delivering post-treatment psychosocial support (the need for trusted healthcare and cultural channels).

**Conclusions:**

Few intervention studies have focused on behaviours among Black CaP survivors, with existing research predominantly involving Caucasian men. There is a need for a collaborative approach to CaP care that recognises not only medical expertise but also the autonomy of Black men as experts of their illness experience, and the influence of cultural and social networks.

## Introduction

With advancements in prostate cancer (CaP) diagnosis and treatment procedures, survival rates for the disease have continued to improve [1]. However, survivors still experience reduced quality of life due to long-term treatment side effects which adversely impact on their physical and psychosocial well-being [2]. CaP treatment side effects may have different implications for Black men due to their disproportionately higher risk (1 in 4) of developing the disease earlier in life, in more aggressive forms and at more advanced stages than Caucasian men (1 in 8) [3].

Despite this poor prognosis, Black men are reportedly less likely to utilise external post-treatment support programmes (e.g., CaP support groups) [4], receive appropriate long-term follow-up care [5] or engage in existing psychosocial interventions as these have predominantly involved Caucasian men [2]. Evidence from research on Black, Asian and Minority Ethnic (BAME) groups highlight correlations between ethnicity and health in which cancer survivors from ethnic minority groups reportedly record a lower uptake of cancer services compared with their majority Caucasian counterparts [6]. Factors which contribute to this disparity are currently not fully understood. However, there are suggestions that preference for alternative coping mechanisms (e.g., religion and spirituality) [7] and perceived lack of cultural sensitivity in patient-healthcare provider communications [8] may be contributory factors.

Previous reviews involving Black men and CaP have mostly focused on screening for early diagnosis [9], knowledge and perceptions [10], post-treatment experiences [11] or quality of life after diagnosis [12] and showed that there are ethnic disparities in how Black men perceive, experience and respond to a CaP illness. Understanding the barriers and facilitators to Black men’s utilisation of psychosocial support after CaP treatment will help to inform the design of useful and acceptable interventions effectively tailored to their support needs and preferences within their sociocultural context. Therefore, this systematic review aimed to synthesise findings from existing published studies on the barriers and facilitators to accessing and utilising post-treatment psychosocial support by Black men after CaP treatment (see operational definition of terms in Table 1).

**Table 1:** Operational definition of terms

**Psychosocial support**: We define psychosocial support as any type of formal or informal but structured non-clinical service, resource, intervention or programme designed to improve men’s psychological, emotional and social well-being after CaP treatment [13]. This includes but not exhaustive to: men and/or couple-focused psychosocial interventions, psychosexual education programmes, peer support, support groups, faith-based groups/organisations, counselling services, information resources (online, face-to-face) and communication activities (e.g., talk with cancer nurse). Personal coping mechanisms (for example, resilience) were excluded because they are perceived to be self-initiated. **Black men**: men of Black African (BA) or Black Caribbean (BC) racial origin, including immigrant and indigenous Black men, and African-American (AA) men	

The Candidacy model [14] which has been widely applied to understand uptake of healthcare services at a broad level [15, 16] is perceived as a useful theoretical framework to enhance conceptual understanding of findings from this review. Postulated by Dixon-woods et al. [14], the Candidacy model articulates how intersections between structural, cultural, organisational and professional factors influence people’s access and utilisation of healthcare services, especially among vulnerable and disadvantaged groups. The authors [17] defined candidacy as “a dynamic and contingent process,” which is iteratively shaped by individual-professionals interactions and the contextual conditions in which those interactions occur (for example, availability of resources). The seven domains of the Candidacy model include identification, navigation, the permeability of service, appearance at health services, adjudications, offers and resistance, and operating conditions [17].

## Methods

### Study design and search strategy

This systematic review is reported following the Preferred Reporting Items for Systematic Reviews and Meta-Analysis (PRISMA) guidelines [18]. The review was conducted using a protocol (PROSPERO registration ID: CRD42020171488) [19] developed following recommended guidelines [20, 21]. Between January and February 2020, systematic searches were conducted on seven databases: Medline, Embase and PsychInfo via OVID, Cochrane database of Systematic Reviews and Central, CINAHL plus and Scopus, from inception to February 2020. The searches were conducted by an experienced information specialist (SG) and librarian (AW), using a validated peer review strategy [22] and search terms iteratively developed from the review aim and PICO (Population, Issue, Context and Outcome) framework for non-intervention studies [23]. Broadly, search terms were words related to: prostate cancer AND psychosocial support AND Black men (20). The search was adapted for each database, with the use of database-specific Thesaurus terms added where appropriate. Boolean operators “AND” and “OR” were used to limit or broaden search results. A supplementary search strategy included searches on OpenGrey, Web of Science proceedings, Google Scholar, Prostate Cancer UK and Movember websites; consultation with professional colleagues; hand-searching of reference list of included articles; and author contact. Search results were managed using bibliographic software: Endnote and Covidence. Database searches were re-run in May 2021.

### Inclusion and exclusion criteria

Primary studies were included if they: (i) involved Black men aged 18 years and above who had undergone active treatment for CaP and (ii) reported on their experiences or suggestions for psychosocial support (as defined above) at the post-treatment phase of their CaP journey. Studies were excluded if they were (i) grey literature which lacked clear methodology (for example, editorials), (ii) conference abstracts whose full papers could not be accessed, (iii) systematic reviews (as focus is on primary studies) or (iv) focused on different ethnic groups and/or cancer types and did not separate the views of Black men after CaP treatment.

### Identification and selection of studies

**Fig. 1:**
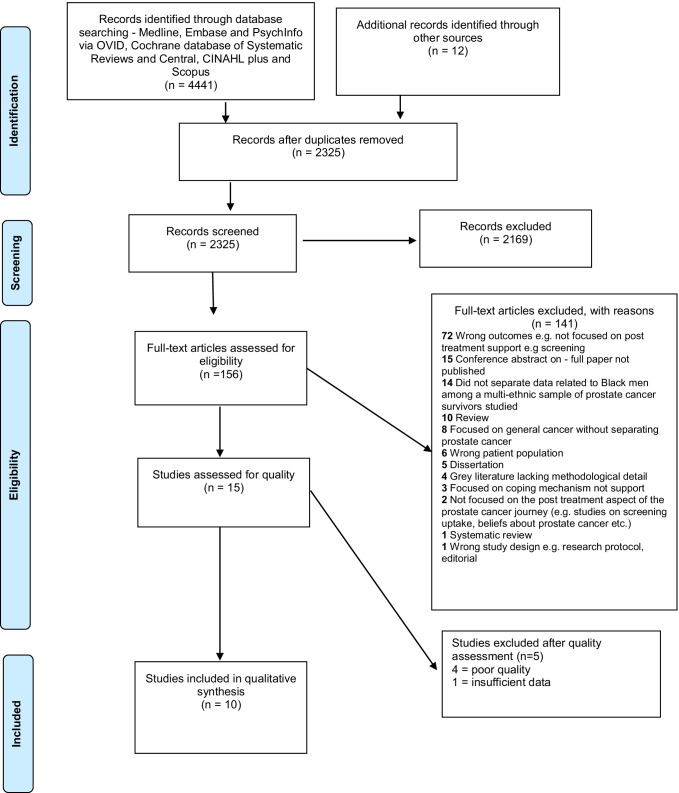
PRISMA diagram

The searches yielded 4453 articles from which 2128 duplicates were removed (Fig. 1). Deduplication was systematically done (by SG) on Endnote and Covidence [24]. Titles and abstracts of the remaining 2325 studies were independently screened for relevance on Covidence [25] and 2169 papers were excluded. Full texts of the remaining 156 studies were screened for eligibility. Fifteen eligible studies were included for quality appraisal. Two reviewers (OB and OA or SG or MO) independently screened the studies and resolved conflicts through discussion.

### Quality appraisal and data extraction

The CASP tool [26] was used to appraise qualitative (*n* = 10) and RCT (*n* = 1) studies whilst the Mixed Methods Appraisal Tool (MMAT) [27] was used for the mixed methods (*n* = 4) papers. Quality score was calculated by dividing “yes” tally by the total number of domains multiplied by 100%. For example, a qualitative study which scored “yes” in nine out of the ten domains on the qualitative CASP tool was scored 90%. Studies were rated as strong (≥ 70%) (*n* = 11), moderate (> 40% < 70%) (*n* = 0) or low (< 40%) (*n* = 4). The mixed methods studies (*n* = 4) were of low quality (17%) and were excluded. Likewise, the only RCT retrieved lacked sufficient data to address the review aim, and was also excluded. Therefore, ten qualitative studies were included in the review, and all were of high quality (≥ 70%) (Table 2). Two reviewers (OB and OA, MO, SG or EDM) independently appraised quality and extracted data using Microsoft Excel (Table 3). Conflicts were resolved by discussion.

**Table 2:** Quality appraisal of included studies using the CASP tool for qualitative studies (CASP 2020)

Study title, authors and year	1. Was there a clear statement of the research aims?	2. Is a qualitative methodology appropriate?	3. Was the research design appropriate to address the aims of the research?	4. Was the recruitment strategy appropriate to the aims of the research?	5. Was the data collected in a way that addressed the research issue?	6. Has the relationship between researcher and participants been adequately considered?	7. Have ethical issues been taken into consideration?	8. Was the data analysis sufficiently rigorous?	9. Is there a clear statement of findings?	10. How valuable is the research?	Total score % = total number of “yes” divided by 10 × 100%
	Yes	Yes	Yes	Yes	Yes	Yes	Yes	Yes	Yes	Yes	
	Can’t tell	Can’t tell	Can’t tell	Can’t tell	Can’t tell	Can’t tell	Can’t tell	Can’t tell	Can’t tell	Can’t tell	
	No	No	No	No	No	No	No	No	No	No	
Bamidele et al. [36]	Yes	Yes	Yes	Yes	Yes	Can’t tell	Yes	Yes	Yes	Yes	90%
Er et al. [34]	Yes	Yes	Yes	Yes	Yes	Yes	Yes	Yes	Yes	Yes	100%
Gray et al. [30]	Yes	Yes	Yes	Yes	Yes	Yes	Can’t tell	Yes	Yes	Yes	90%
Hamilton et al. [29]	Yes	Yes	Yes	Yes	Yes	No	Yes	Can’t tell	Yes	Yes	80%
Imm et al. [35]	Yes	Yes	Yes	Yes	Yes	No	Can’t tell	Yes	Yes	Yes	80%
Jones et al. [31]	Yes	Yes	Yes	Yes	Yes	Yes	Yes	Yes	Yes	Yes	100%
Margiriti et al. [37]	Yes	Yes	No	Yes	Yes	No	Yes	No	Yes	Yes	70%
Nanton and Dale [32]	Yes	Yes	Yes	Yes	Yes	Yes	Yes	Yes	Yes	Yes	100%
Rivers et al. [33]	Yes	Can’t tell	No	Yes	Yes	Yes	Yes	No	Yes	Yes	70%
Wagland et al. [38]	Yes	Yes	Yes	Yes	Yes	Can’t tell	Yes	Yes	Yes	Yes	90%

**Table 3:** Summary of studies included

Authors, year of publication and country	Study aim	Study design (plus analytic method if stated)	Quality appraisal score	Sample characteristics	Treatment side effects	Experience of psychosocial support	Barriers to access/use of psychosocial support	Facilitators to access/use of psychosocial support	Key conclusion/recommendations
Bamidele et al. 2019 (UK)	To explore the psychosocial experiences of BA/BC men with prostate cancer and their partners in the United Kingdom as they lived through the side effects of treatment within their own sociocultural and marital contexts	Qualitative study using semi-structured interviews and focus groups (grounded theory)	90%	25 men: BA (8) BC (17) treated for prostate cancer; length of time since treated — 3 months ≥ 10 years; 11 female partners and 11 HCPs. The men were aged between 45 and 88 years, treated with surgery (*n* = 8), brachytherapy (*n* = 5), radiotherapy with hormone treatment (*n* = 7), surgery with radiotherapy (*n* = 3), hormone treatment with chemotherapy (*n* = 1) and hormone treatment with cryotherapy (*n* = 1). Married (*n* = 19), single (*n* = 1), widowed (*n* = 1) and unspecified (*n* = 1).	Sexual dysfunction, urine incontinence	Support from: partners and immediate family (children); HCPs, peers who had experienced similar prostate cancer journey; employers; pastor and church friends (offered prayers and encouragement)	(i) Lack of psychosexual support service within post-treatment healthcare provision; (ii) reluctance towards public disclosure of the prostate cancer; (iii) cultural masculinity values to be independent and “macho”; (iv) having a solo approach towards dealing with post-treatment issues; (v) dismissive behaviour by HCPs	Personal faith and spirituality/religion made the men receptive towards support from their church community	(i) Psychosocial intervention should be delivered in a way that promotes self-management of symptoms, and as part of routine post-treatment prostate cancer care
Er et al. 2017 (UK)	To explore the barriers and facilitators to dietary and lifestyle changes and the acceptability of a diet and physical activity intervention in African-Caribbean prostate cancer survivors	Qualitative study using semi-structured interviews (thematic analysis)	100%	14 African-Caribbean men treated for prostate cancer, time since diagnosis < 5 years. Treated with radiotherapy with hormone therapy (*n* = 9) or prostatectomy (*n* = 3); length of time since treated not reported; aged 52–80 years, median age 71.5 years. Married or living with partner (*n* = 10); single or widowed (*n* = 4).	Incontinence and erectile dysfunction	Identified partner role in providing support by implementing dietary changes	(i) Lack of personal conviction in intervention benefit; (ii) physical health limitations (e.g., treatment-related incontinence; old age) and sports injuries; (iii) wanting to “move on” and not dwell on the cancer; (iv) personality type, e.g., sanguine; (v) financial challenges brought about by the prostate cancer diagnosis, e.g., loss of income; (vi) reluctance to give up usual routine habit or try something new; (vii) preference for individual versus group-based exercise; (viii) low awareness of support services	(i) Perceiving intervention as a catalyst for desired change; (ii) advice from HCPs who men perceived as experts and the ideal source of trusted information; (iii) perceived benefit of interventions to reduce PSA level; (iv) self-motivation to deal with treatment side effects without relying on medications; (v) influence of partners and members of men’s social support network as “change agents”; (xii) personal autonomy to choose how to engage with interventions, e.g., preference to walk alone, at own time and pace, instead of in a group	(i) Information on interventions to be given by HCPs; (ii) intervention should be matched to men’s age and what their physical strength can accommodate; (iii) interventions with clinical impact — targeted at reducing PSA level; (iv) interventions which emphasise/promote personal autonomy and responsibility; (v) development of a coherent model of referral structure/pathway to lifestyle services and health and well-being
Gray et al. 2005 (Canada)	To reveal how prostate cancer affects the lives of individual Black men and to show how a narrative approach contributes to health psychology	Qualitative study using open-ended interviews (narrative approach)	90%	Two Black men (one from the Caribbean, the other Canadian of Caribbean origin? not clear) treated for prostate cancer; treatment type: radiotherapy and surgery; length of time since treated: Paul — 1-year post-surgery, John — not reported; aged > 70 years and 62 years, respectively. Both married.	Sexual dysfunction — partial and total loss of sexual function; urine incontinence	Local prostate cancer support group where men had access to helpful information and peer support; support from partner, friends and employer	Not reported	(i) Access to helpful information; (ii) presence/support of peers with shared experiences and concerns in the support group; (iii) having close relationship with peers with similar experience and who show respect for own spiritual work	(i) Inclusion of wives in psychosexual advice — doctor to tell wife to take leading role in sexual activity and help husband navigate erectile dysfunction
Hamilton and Sandelowski 2004 (USA)	To describe the types of social support that African-Americans use to cope with their experience of cancer	Qualitative study using open-ended and semi-structured interviews (grounded theory)	80%	13 African-American men treated for prostate cancer; length of time since treatment not reported; aged 61–79 years. Average age was 67 years. The majority were married (*n* = 11), well-educated and retired (*n* = 10)	Not reported	(i) Emotional support (presence of others especially partner, family, friends); (ii)support from the church in form of prayers, assistance to continue to maintain religious practices and role; (iii) information from peers with similar illness experiences to validate information received from HCPs	Not reported	(i) Continued maintenance of religious and social roles in the family, workplace and church	(i) Dissemination of information through informal networks especially peers with similar illness experience
Imm et al. 2017 (USA)	To explore the prostate cancer survivorship experience of African-American men and the potential unique factors that contribute to quality of life outcomes among African-American survivors	Qualitative study using focus groups (thematic analysis)	80%	12 African-American men treated for prostate cancer; treatment type: radical prostatectomy (open surgery—5; robotic surgery—7); length of time since treated 7–31 months, mean 19.8 months; aged between 49 and 79 years, mean age 61 years; married (*n* = 2); never married/divorced/widowed (*n* = 5); marital status unknown (*n* = 5)	Urinary problems, sexual dysfunction; post-surgical weakness	Organised social support group (The Empowerment Network or TEN); hospital prostate support group. Support mostly received from partner, family and friends	(i) Preference for non-health profession social support networks (e.g., community, friends and family); (ii) upholding traditional masculinity norms to be “macho”; (iii) cultural norm to keep illness private	(i) Presence of peers with shared experience in the support group	(i) Inclusion of fitness program in support group activities to encourage communication and support seeking
Jones et al. 2011 (USA)	To explore cancer support and financial issues related to cancer care experienced by African-American men with prostate cancer and to understand whom they relied on for resource issues during diagnosis and treatment	Qualitative study using focus groups (thematic analysis using hermeneutic phenomenology)	100%	23 African-American (11 rural:12 urban) men treated for prostate cancer; treatment type: radiation treatment (*n* = 16), prostatectomy (*n* = 6); radiation + hormonal therapy (*n* = 2); prostatectomy + radiation (*n* = 3); hormonal therapy (*n* = 2); length of time since treated not reported; aged between 66 and 80 years, mean age of 73 years. Married (*n* = 14); widowed (*n* = 2); single (*n* = 3); divorced or separated (*n* = 3); living with partner (*n* = 1)	Not reported	Experienced: support from church community through prayers. Support mostly received from partner, family and friends	(i) Lack of affordability of healthcare if not have medical insurance; (ii) geographical location (living in a rural area)	(i) Personal faith and spirituality/religion; (ii) having healthcare insurance; (iii) geographical location (living in an urban area)	(i) Improving access of African Americans to healthcare insurance; (ii) using community health workers with shared characteristics to deliver psychosocial intervention (informal counselling, social support and health education)
Margiriti et al. 2019 (UK)	To understand the experiences of African-Caribbean men with respect to their discharge to primary care following successful prostate cancer treatment and the challenges associated with survivorship	Qualitative study using a focus group (thematic analysis)	70%	8 African-Caribbean men treated for prostate cancer; treatment type: radiotherapy, surgery; length of time since treated not reported; aged 55–75 years. Married (*n* = 4), single (2), widowed (*n* = 1), cohabiting (*n* = 1)	Urine incontinence, loss of sexual potency	Accessed support from Primary Care (GP); attendance at support group which was perceived as a safe place to receive information, comfort, counselling and mutual sharing of worries and concerns	(i) Lack of psychological support to complement clinical care received at primary care; (ii) GPs lacking knowledge on greater risk of prostate cancer among Black men leading to inadequate provision of support; (iii) Black men’s reluctance to ask for support because of the need to retain their masculine ideals to be sexually potent as prescribed by cultural stereotypes and expectations; (iv) having to pay for sexual aids; (v) perceptions that disclosure of concerns is not consistent with masculinity norms as Black men	(i) Perception of support group as a safe place to receive information, comfort, and can openly share worries and concerns; (ii) feeling comfortable with other members of the support group because they share a similar experience and have mutual understanding of each other’s feelings	(i) Provision of counselling and psychological support for erectile dysfunction within post-treatment clinical care; (ii) continuity of care with same GP whom men are already familiar with and built trust with to share their sensitive concerns; (iii) training of GPs and healthcare professionals on cultural sensitivity to the social and emotional needs of Black men
Nanton and Dale 2011 (UK)	To identify whether and in what way ethnicity played a distinctive role in determining the disease and healthcare experiences of first-generation African-Caribbean men with prostate cancer	Qualitative study using interviews (thematic analysis)	100%	16 African-Caribbean (Jamaican) men treated for prostate cancer; time since diagnosis ranged from 1 to 20 years, median time since diagnosis is 2 years/treated by prostatectomy (*n* = 8), or hormones (*n* = 2) or radiotherapy with hormone (*n* = 2) or prostatectomy + radiotherapy + hormone (*n* = 1) or catheter only (*n* = 1) or watchful waiting (*n* = 1). Length of time since treated not reported; aged between 50 and 83 years, median age 72.5 years. Married (*n* = 11), widowed (*n* = 5) years	Urinary incontinence, sexual dysfunction; post-surgery pain	Support mostly received from partner, family and friends; sources of external support; hospital prostate club, local advice agencies, informal support networks with other local men with prostate cancer, encouragement from the church; perceived services from local advice agencies as inadequate and inappropriate; unsatisfactory post-treatment support from hospital.	(i) Lack of awareness of psychosocial support services; (ii) perceived difficulty accessing the service (where sign-posted), e.g., having to make several phone calls; (iii) complex referral procedures, e.g., having to refer self; (iv) content of service provided not suitable to meet men’s needs; (iv) services perceived as inadequate or inappropriate	(i) Support delivered by the church community; (ii) referral by healthcare professionals; (iii) being physically active	(i) Inclusion of church leaders in counselling and support interventions developed by HCPs for African-Caribbean men; (ii) HCPs to be proactive in eliciting and providing information; (iii) establishment of systematic links, referral procedures and information exchange between clinicians, social care agencies and community organisation (including the church); (iv) training of HCPs on cultural sensitivity to the needs of African-Caribbean men, including patient-healthcare provider communication that is culturally appropriate
Rivers et al. 2012 (USA)	To explore the perceptions of African-American prostate cancer survivors and their spouses of psychosocial issues related to quality of life	Qualitative study using semi-structured interviews (thematic analysis based on Ferrell’s conceptual model of QOL)	70%	12 African-American men (and their wives) treated for prostate cancer; treatment type: surgery, radiotherapy (including brachytherapy), and surgery + radiotherapy. Length of time since treated not reported; length of time since diagnosis: 2–5 years; married between 5 and 46 years. The men were aged 51–70 years with a mean age of 59.75 years.	Erectile dysfunction, incontinence, fatigue, difficulty/pain when urinating; fluctuating weight, lack of appetite	Virtual support groups on the internet; peer support; support from the church; support mostly received from partner, family and friends	(i) Lack of information; (ii) non-availability of counselling support	(i) Desire for information to deal with a scary illness; (ii) personal faith and spirituality	(i) Information and counselling to manage erectile dysfunction; (ii) spiritual counselling and faith-based interventions; (iii) dissemination of salient information on symptom management and effective spousal communication strategies through culturally tested channels and context; (iv) intervention to include family-focused resources to help enhance communication between couples
Wagland et al. 2020 (UK)	To explore adjustment strategies adopted by Black African and Black Caribbean men in the UK as a response to the impact of prostate cancer diagnosis and treatment side effects	Qualitative study using semi-structured telephone interviews (framework analysis)	90%	14 African-Caribbean men (10 BAs, 4 BCs) men treated for prostate cancer; treatment type: radical prostatectomy ± (*n* = 6), radiotherapy + ADT (*n* = 4), ADT only (*n* = 1), active surveillance/watchful waiting (*n* = 2), chemotherapy ± other (*n* = 1) length of time since treated not reported; aged between 55 and 85 years, mean age 66 years. Married (*n* = 9), single (*n* = 5).	Urine incontinence; erectile dysfunction; decline in body image; weight gain	Sources of support: support group sign-posted by HCPs; community support group linked to cultural identity; church support; perceived support and information received from the NHS as “fantastic”; majority of support from partner, immediate family, friends and the church	(i) Need to maintain a positive front that “all is well with me”; (ii) social stigma associated with illness disclosure; (iii) being too shy; (iv) reluctance to discuss the prostate cancer diagnosis; (v) not needing support (self-reliance)	(i) Sign-posting to support services by HCPs; (ii) existing affiliations with religious or cultural community groups; (iii) presence of others with shared experience in the group	Use of culturally matched local peer champions (those who had undergone similar illness experience) to pitch information messages and support

### Data analysis

We analysed data using thematic synthesis [28] which involved three stages. Firstly, we freely coded findings from each individual study using words directly from the reported data (where possible). We then aggregated similar codes into descriptive themes using labels which allowed us to stay close to the original data as much as possible. Lastly, we generated new analytical constructs by exploring similarities, differences and patterns in the descriptive themes and interpreting these in relation to the review aim. The new analytical themes were then labelled accordingly. Two reviewers (OB and AW) independently analysed data and deliberated developed themes with another two reviewers (OA and EDM). Data analysis was managed with NViVo 12 software.

## Results

### Overview

**Fig. 2:**
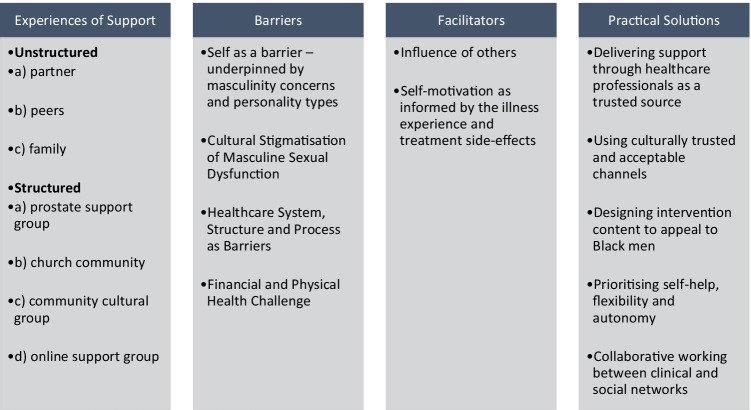
Overview of analytical constructs and associated descriptive themes

The ten qualitative studies [29–38] were published between 2004 and 2020 and conducted in the UK [32, 34, 36–38], USA [29, 31, 33, 35] and Canada [30]. A total of 139 Black men (60 AA, 60 BC, 18 BA and 1 unspecified) aged between 49 and 85 years were included in the studies (Table 3). Whilst the studies were all qualitative, data collection and analytic methods varied as shaped by their respective study designs (Table 3). Data analysis yielded four analytical constructs (Fig. 2): “experience of psychosocial support for dealing with treatment side effects,” “barriers to use of structured post-treatment psychosocial support,” “facilitators to use of structured post-treatment psychosocial support” and “practical solutions for designing and delivering structured post-treatment psychosocial support.”

### Experience of psychosocial support for dealing with treatment side effects

A predominant theme across all but two [29, 31] of the studies was the psychological impact of treatment side effects (e.g., sexual dysfunction) on the men. Men expressed feelings of stress [32, 33, 35]; discouragement [30, 32, 36]; isolation from social contacts [32]; injured self-esteem (due to a restricted ability to perform their daily routines) [32, 35]; marital insecurities [35]; fear of cancer reoccurrence or fatality [33, 37]; and diminished masculinity [30, 33, 36] after treatment (Table 4 (1i)). Although desiring more information from their healthcare providers to manage these challenges, men recounted positive experiences of receiving unstructured practical and emotional support from their partner, family and wider social networks, including peers who had undergone a similar illness experience. These social networks supported the men through prayers, information provision, moral support and encouragement. In all ten studies, partners were unanimously highlighted as the main source of support for the men, through emotional and practical help, and enabling lifestyle changes where necessary (Table 4 (1ii–iv)).

**Table 4:** Themes and supporting quotes

Themes	Supporting quotes from the included studies
1. Experience of psychosocial support for dealing with treatment side effects	*i“…The only problem I have is it seemed like it makes me less than what I thought I was. I mean, being a male, you know, you have these male tendencies to think that you have all, everything and with the prostate cancer … and see, mine was a major surgery; they took everything so all I have left was just my … my … my regular male genders but there’s no function; I mean, there’s no … you can’t get an erection on your own and this kinda things; you have to have help to do that and that’s the only real part … major part I have problems with…” (Participant quote *[33]*)* *ii“… more or less it’s the wife that is leading the process (dietary change) really. (Laugh) Yeah, she’s the one doing the shopping, she’s the one providing the food. And now she’s, she’s going and, and do all the shopping and buy whatever she thinks that’s good for our health…” (Participant quote* [34]*)* *iii“…In the period following treatment, John’s wife supported him in practical ways, cooking and caring for him, helping him deal with temporary incontinence…” (Participant quote* [30]*)* *iv“…I would say my support group pretty much is my family. They just took care of me…” (Participant quote* [35]*)* *v“…Well, apart from wife, my church stands by me with my illness and my friends. Everybody who knew that you were ill will encourage you and that is the help I get. I didn’t get it from any (other) organisations…” (Participant quote* [32]*)* *vi“…We have a [West African Country] Community Association and we meet up regularly …and discuss issues, for example issues on prostate cancer, diabetes, on health issues in general. … we have three or four who have had [PCa] and who have gone successfully for the treatment. We meet up and have a chat and a discussion about it...” (Participant quote* [38]*)*
2. Barriers to use of post-treatment psychosocial support	
a. Self as a barrier — underpinned by masculinity concerns and personality types	*i“…So, so what the difference that it makes to, it’s not going to affect me now because I’m, I’ve [passed that stage] with the prostate cancer…” (Participant quote* [34]*)*
b. Healthcare system, structure and process as barriers	*i“…I don’t know who to go to for help here…” (Participant quote* [32]*)* *ii“…(I went to see) the welfare rights and she was explaining to me that I could get help for certain things, but . . . like, ‘Why don’t you try the Macmillan ...?’ But by the time you’re phoning here and phoning there and phoning there, I said ‘I can’t worry about it…’” (Participant quote* [32]*)* *iii“…I am going to my GP every 6 months to have my PSA but…no one asked me “how are the things with you? How you are feeling?” there is no occasion about that and I am going through this since 2008…” (Participant quote* [37]*)* *iii“…The majority of the survivors reported that they were not provided with counselling or in-depth information on possible options to manage ED…” * [33]
c. Cultural stigmatisation of masculine sexual dysfunction	*i“…Oh no because you don’t talk about erectile dysfunction and black men, no, you don’t talk about it…” (Participant quote* [35]*)* *ii“…not something in our culture that we normally dwell on very much. To us as a man, it’s dehumanising isn’t it. It’s not something that you can really talk about very much…”. (Participant quote* [38]*)* *iii“… but Black men I think sexually tend to be quite private, erm we struggle to even have discussion with our peers about prostate cancer, … it’s a stigma sort of thing you know …” (Participant quote* [36]*)*
d. Financial and physical health challenges	*i“…because I’m very concerned about the African American people…we have to do something about the number of people in this country who don’t have health insurance… That’s a gap right there. They don’t have the finances…” (Participant quote* [31]*)* *ii“…I want to go to the gym soon, but like I said, because I’m so wet I won’t have – I’m hoping that it will ease up a little bit so I can get to do something else…” (Participant quote* [34]*)* *iii“…I’m too old to go to the gym man. I don’t want to go to the gym to get a heart attack. (Laughter)…I used to go on it but I don’t want to do all this type of things now…” (Participant quote* [34]*)*
3. Facilitators to use of post-treatment psychosocial support	
a. Influence of others	*i“… Fantastic. The support and information I got from the NHS was really, really good…” (Participant quote* [38]*)* *ii“…the main benefit was the sense that others shared his battle to overcome feeling ‘like a discard’ (‘I realize now that I’m not the only catfish in the sea’) …” (Participant quote* [30]*)* *iii“…the TEN network which is an African-American support group…They were very supportive and connected me with guys who have gone through this before me…” (participant quote* [35]*)* *iv“…There are so much knowledge and information and you feel so comfortable around the group because you know that they’ve gone through similar and you have that degree of understanding as well, cause your people can be understanding at a certain level but they have not been through it, you know…” (Participant quote* [37]*)*
b. Self-motivation as informed by the illness experience and treatment side effects	*i“…You’ve got a bigger belly than you had before, and if you want- what do you want to get out of this?’ And I said, ‘I’d like to probably get my shape back, and just lose a little bit of weight…’ (Participant quote* [34]*)* *ii“…so, I’ve got no qualms about speaking about it (prostate cancer diagnosis) …” (Participant quote* [38]*).* *iii“…Both the survivors and spouses desired more information and guidance on techniques and resources to assist in the management of the effects of treatment on sexual functioning…” (* [33]
4. Practical solutions for designing and delivering post-treatment psychosocial support	
a. Delivering support through healthcare professionals as a trusted source by default	*i*“…*she [dietician] said with, with the prostate cancer that I’ve got I should eat a lot of tomato. Tomato is good for the prostate cancer. My doctor before, Doctor M, told me that if I drink pomegranate juice it’s a little bit helpful as well…That’s what I buy, we buy, things, we buy pomegranate juice...” (Participant quote* [34]*)* *ii“…Our study more specifically suggests that the experience of African-Caribbean men with prostate cancer would be improved through a more proactive approach to eliciting as well as giving information by healthcare professionals…” (Authors’ recommendation* [32]*)* *iii“…He (the doctor) should have called her (patient’s wife) into the office and said, these are your new responsibilities, stick to them and it would help, and these are the reasons why it would help, and be beneficial in the end…” (Participant quote* [30]*)*
b. Using culturally tested and acceptable channels	*i“…This study suggests local champions may also ‘pitch’ information in a culturally sensitive way...” (Authors’ recommendation* [38]*)* *ii“…spiritual counselling was suggested by some participants as a valuable service for both cancer survivors and their spouses…” (Participants’ recommendation* [33]*)*
Prioritising self-help, flexibility and autonomy	*i“…Na, because again I have to, they want, when I’m ready to do it they won’t be ready, that’s the problem. That’s why I have to do my own…” (Participant quote* [34]*)*
d. Collaborative working between clinical and social networks	*i“…establishment of systematic links, referral procedures and information exchange between the GP, hospital, statutory social care agencies, the voluntary sector, local churches and community organisations would play an important role in improving the long-term quality of life of the most vulnerable African-Caribbean men with prostate cancer…” (Authors’ recommendation* [32]*)*
e. Designing intervention content to appeal to Black men	*i“…Therefore, a dietary and physical activity intervention which enhances men’s autonomy, framed as helping men to regain fitness and aid post-treatment recovery and is aimed at men with elevated PSA may be appealing and acceptable to African Caribbean prostate cancer survivors...” (Authors’ recommendation* [34]*)* *ii“…Most men (aged 70 and above) viewed themselves as too old to be playing sports and gentle exercise as safer and more appropriate for their age. Therefore, a brisk walking intervention was perceived as safe and acceptable by men in the study…” (Participant quote* [34]*)*

Men also accessed structured support from local church communities [29, 31, 32, 36, 38] (*n* = 6), prostate cancer support groups [30, 32, 35, 37, 38] (*n* = 5), cultural community groups [32, 38] (*n* = 2) and an online support group [33] (*n* = 1). Support from local church communities was offered through communal prayers, and spiritual encouragement, which men reported as valuable (Table 4 (1v)). Men who attended a prostate cancer support group described it as a “*safe place where they could find information, comfort, exchange thoughts and be open about their concerns and worries*” [37]. Two studies [32, 38] reported men also accessed support through participation in health talks at cultural community groups where they had opportunity to discuss with other survivors who were ahead of them in the CaP journey (Table 4 (1vi)). Only one US-based study [33] reported men accessing information through a virtual support group on the internet.

### Barriers to use of structured post-treatment psychosocial support

#### These are reported under four descriptive themes.

##### Self as a barrier — underpinned by masculinity concerns and personality types

Men reported exercising personal autonomy to decide their uptake of structured psychosocial support. Personality types (e.g., being a private person or being too shy) [33, 35, 37] and masculinity concerns [30, 32, 33, 35, 36] around losing their independence, self-esteem and to “*avoid being perceived as weak*” substantially influenced men’s reluctance to access structured psychosocial support. Using personal coping strategies such as resilience [32, 34, 36] and self-reliance [33, 34, 36, 38], and lacking conviction in intervention benefit [34], were additional barriers to men’s engagement with structured psychosocial support (Table 4 (2ai)).

##### Cultural stigmatisation of masculine sexual dysfunction

In some studies [35, 36, 38], men were reluctant to access structured psychosexual support in order to avoid perceptions of diminished masculinity often associated with post-treatment sexual dysfunction within their cultural setting. Men avoided public disclosure and highlighted that their CaP was not a subject for discussion at a social level due to concern that they would be perceived as effeminate within their cultural circle (Table 4 (3ai–iii)).

##### Healthcare system, structure and process as barriers

Findings showed that men perceived their routine CaP care is predominantly focused on clinical management of physical side effects of treatment, with minimal or no provision for structured psychosocial support. Some studies [32, 33, 36, 37] (*n* = 4) reported men were neither informed nor sign-posted to appropriate psychosocial support by healthcare professionals (HCPs) which meant they were not aware of existing services (Table 4 (2bi)). In one study [32] where men were sign-posted to support services, they reported that complicated referral procedures made it difficult for them to access such services (Table 4 (2bii)). Men highlighted a lack of psychosexual services (e.g., counselling) to help them deal with the psychological impact of sexual dysfunction (Table 4 (2biii–iv)). A few studies [32, 37] highlighted racial stereotyping of Black men’s sexuality by doctors (stereotypical comments from doctors on Black men prioritising their sex life over dealing with the CaP illness), which prevented men from openly expressing their need for psychosexual support during clinical consultations.

##### Financial and physical health challenge

Findings from a few studies [31, 34, 37] showed that financial challenges somewhat limited men’s uptake of structured psychosocial support. For example, some men [34] reported loss of income due to their CaP diagnosis. Thus, they prioritised returning to work over participation in a lifestyle intervention designed to help men deal with CaP treatment side effects. Men in a US-based study [31] highlighted lack of health insurance as a barrier to healthcare access among AA men (Table 4 (4i)). Physical limitations from old age, injuries from sports and other health challenges (e.g., from urinary incontinence) were additional barriers to men’s participation in physical activity intervention for CaP survivors [34] (Table 4 (4ii–iii)).

### Facilitators to use of structured post-treatment psychosocial support

#### Facilitators were reported under two descriptive themes.

##### Influence of others

This finding emerged from all of the studies although from different perspectives. A few studies reported that men received support from their HCPs via information provision [36, 38] or sign-posting to hospital prostate support groups and social support agencies [32]. However, the psychosocial support men received from their HCPs appeared to be blurred along the different stages of the CaP spectrum as there was a lack of clarity on which support was received at the post-treatment phase of the illness journey (Table 4 (3ai)). Men who attended prostate support groups recalled receiving information and motivation to attend from their peers who were members of the group and had a similar CaP experience [30, 35, 37, 38] (Table 4 (3aii–iv)). In one of the studies [34], men narrated their partner influenced them to adhere to a lifestyle intervention which involved dietary changes for healthier living.

##### Self-motivation as informed by the illness experience and treatment side effects

Some studies [30, 33, 34, 38] reported men were self-motived to use developed psychosocial interventions because they perceived them as complementary to their existing dietary lifestyle or an indication of recovery from the CaP. Men reiterated a personal decision to regain their pre-diagnosis body shape and fitness naturally without dependence on medications, thus, they engaged with developed lifestyle interventions (Table 4 (3bi)). Some men viewed their CaP diagnosis as a stimulant to make necessary lifestyle changes they had always wanted to make, particularly as they grew older (for example, making dietary changes and engaging in CaP advocacy activities) (Table 4 (3bii)). Men acknowledged a significant impact of treatment-related sexual dysfunction on their sex lives and psychosocial well-being [33, 36] and had expectations for professional psychosexual support to be delivered as part of routine post-treatment CaP care (Table 4 (3biii)).

### Practical solutions for designing and delivering structured post-treatment psychosocial support

Review findings identified some practical solutions for designing and delivering psychosocial support for Black men after CaP treatment. We also considered individual study authors’ recommendations (where linked to their participants’ narratives) as they offered additional useful insights which complemented the men’s suggestions. These are reported under five descriptive themes.

#### Delivering support through healthcare professionals as a trusted source

Perceiving HCPs as a skilled and trusted source of credible information, findings indicated Black men may be willing to engage with developed psychosocial interventions if they are either delivered or sign-posted by their HCPs [30, 32, 34, 36] (Table 4 (4ai–ii)). For psychosocial support delivered by HCPs, men suggested partners should be included and HCPs should prescribe their role in initiating sexual activity. Men explained this could help facilitate mutual problem-solving for sexual problems among couples, and reduce the psychological burden of sexual dysfunction on the men (Table 4 (4aiii)).

#### Using culturally trusted and acceptable channels

Review findings [29, 31–33, 38] showed that delivering psychosocial support through culturally acceptable and trusted avenues could potentially improve Black men’s use of such support as this will help to situate their illness experiences within their familiar setting and improve culturally sensitive cancer care for them. Some studies suggested the use of relatable local community champions or peers with shared ethnicity whom men can identify with, to provide peer support and information (Table 4 (4bi)). Reflecting on spirituality and faith beliefs as their key coping strategies, some men suggested the incorporation of faith-based interventions (e.g., counselling services and health education) in psychosocial support for CaP survivors within the Black community (Table 4 (4bii)).

#### Designing intervention content to appeal to Black men

Review findings showed that the content of developed interventions needs to be appealing to Black men, in order to stimulate their interest and engagement. Men highlighted the need for psychosocial support to achieve visible clinical impact (e.g., reduced PSA levels and regaining of pre-cancer body weight) and be targeted at allaying their fears of cancer reoccurrence (Table 4 (4ei)). Men also seemed to prefer psychosocial support which promotes independence, factual information, practical resources and couple-focused activities to enhance communication and mutual problem-solving. For group-based support programmes, it was suggested this should take into account disparities in men’s demographics so that men can identify with their counterparts from a similar age group (Table 4 (4eii)). Findings [29, 30, 32, 37] highlighted the need for improved cultural competence and sensitivity among HCPs when delivering psychosocial support to Black men with CaP by demonstrating a contextual understanding of the dynamics of masculinity within the Black cultural group and how these mediate their help-seeking for sexual problems after CaP treatment.

#### Prioritising self-help, flexibility and autonomy

Findings highlighted the need to consider Black men’s priority to retain their idealistic masculine identity when developing post-treatment psychosocial support for them. For example, there were suggestions that support programmes which prioritise men’s independence and personal autonomy [34] and are delivered to promote “*self-management of sexual problems without appearing emotionally weak*” [36] may be more appealing to Black men with CaP. In particular, men narrated the need for support programmes to be flexible and allow them to choose which aspects of the programme they would like to engage with, in their own time and at their own pace (Table 4 (4ci)).

#### Collaborative working between clinical and social networks

Study authors [29, 31, 32, 34] recommended the establishment of strategic links, referral procedures and information exchange between clinicians, social care agencies, religious and community organisations in developing and delivering affordable structured psychosocial support and CaP education programmes for Black men. The perception is that such collaboration could help to increase awareness and take-up of available support services by Black men if such information is disseminated at the grassroot level (Table 4 (4di)). All supporting quotes are presented in Table 4.

## Discussion

This systematic review synthesised findings from existing published studies on the barriers and facilitators to accessing and utilising post-treatment psychosocial support by Black men after treatment for CaP. Some findings from this review resonate with evidence from studies on Caucasian men where practical issues [39], privacy concerns and lack of awareness of services [40] were likewise reported as barriers to men’s attendance at CaP support programmes. However, the current review has identified additional factors which are relevant to Black CaP survivors and which challenge assumptions that this population do not access external psychosocial support services predominantly because of an ingrained aversion towards public disclosure of their illness [4, 5]. Rather, there are wider factors which intersect between cultural, structural/organisational, personal and social factors, which influence Black men’s access and utilisation of organised psychosocial support programmes. This supports postulations from the Candidacy model [17] that the intersection of such factors impacts on people’s access and utilisation of healthcare services, especially among vulnerable and disadvantaged groups.

The theme around “self” as a barrier and facilitator to accessing and utilising psychosocial support in this review reinforces the centrality of personal autonomy and independence to how Black men with CaP perceive, navigate and respond to support services along their post-treatment journey [41, 42]. The “identification” and “appearance at health services” constructs of the Candidacy model postulate that an individual’s recognition of their need to seek help for symptoms they are experiencing, and their ability to articulate what help they require, substantially influence their uptake of healthcare services. Moreover, the masculine ideology of being in charge of key decisions as portrayed by men in the studies included in this review hints at the importance of viewing them as partners in developing psychosocial support through mutual decision-making and coproduction [43].

Using a conceptual model of coproduced healthcare, Batalden et al. [44] postulate that participatory interactions between patients and HCPs within their societal healthcare system are influenced by “*the structure and function of the healthcare system*” and wider social services. Men’s expectation in this review, of HCPs providing or sign-posting them to post-treatment psychosocial support, highlights the centrality of their role to the men’s illness journey. The Candidacy model [17] recognises HCPs as adjudicators of healthcare access with an important role to provide patient-centred support services (Adjudication), make patients aware of (Navigation) and facilitate their attendance (e.g., through referrals) at such services (Permeability of service). Prioritising self-help, flexibility and provision of factual information could further make the “operating conditions” of developed interventions appealing to Black men [17, 45]. Whilst there is an increasing use of online support among Caucasian men with CaP [46], review findings suggest this may be less appealing to Black men as only one study [33] reported men accessed online support groups. Fogel et al. [47] identified digital inequality, cultural preferences, trust issues and preference for face-to-face support as key barriers to engagement of African-Americans (AAs) in online cancer support groups.

### Implications for practice

Whilst men mostly demonstrated personal autonomy in decision-making for their support preference, the influence of social networks (e.g., partners, peers and religious communities) on their help-seeking behaviour towards psychosocial support cannot be ignored. This suggests the potential benefit of a collaborative approach to CaP care which recognises the autonomy of men as experts of their illness experience, medical expertise of HCPs and influence of social networks on men’s help-seeking for post-treatment support. The use of culturally acceptable channels (e.g., partners and religious leaders) to mediate health behaviour change in Black men with CaP is well recognised in the literature [4, 48, 49] and should be adopted when developing post-treatment psychosocial support services for them. Given the increasing digitalisation of healthcare service delivery (including cancer services) [50] facilitated by rapid technological advancements and the COVID-19 pandemic, it is important for HCPs to consider the reluctance of Black men towards online support. Hence, they should highlight to men, the benefit of online support to promote flexibility and autonomy in health service delivery. Online support resources should also be intuitive, simple and interactive whilst prioritising patient’s data protection and safety online. Wider evidence on BAME groups [51, 52] suggest they may be receptive towards online interventions if accessible on mobile devices, and complemented with professional support.

### Study limitations and directions for further research

This is the first systematic review to the best of our knowledge, which synthesised data from individual qualitative studies to produce a more in-depth conceptual and contextual evidence on barriers and facilitators to accessing and utilising structured psychosocial support by Black men after CaP treatment. However, this review has some limitations. Studies included were conducted in three different countries (UK, USA, and Canada) with disparities in healthcare structures and systems. Therefore, men’s respective contexts should be considered when interpreting review findings. For example, financial challenge was reported as a barrier in both US and UK studies but with differing perspectives. It was reported as a health insurance problem for US-based men, but expressed as inability to return to work quickly in the UK. This highlights the complexity of delineating diversities in the support priorities of CaP survivors despite having shared racial backgrounds. Most of the included studies did not provide details on length of time since the men were treated for CaP, which makes it difficult to understand how their support needs may have evolved through the post-treatment phase of their illness trajectory. Further research is needed to investigate this phenomenon. Search results showed a dearth of psychosocial intervention studies focused on behavioural issues among Black CaP survivors as these have predominantly involved Caucasian men [2, 53]. This important gap in the literature warrants further research.

## Conclusions

This study is an incremental addition to the extant literature as it explores additional domains that were relatively unknown about psychosocial support utilisation among Black men after CaP treatment. This study is novel in that it: (1) showed that intersections between cultural, structural/organisational, personal and social factors influence access and utilisation of structured post-treatment psychosocial support services by Black CaP survivors; (2) highlighted the relevance of the Candidacy model for use in CaP research (beyond its use in healthcare services); and (3) highlighted unique factors (both barriers and facilitators) relevant to Black CaP survivors regarding their access to and uptake of psychosocial support post-treatment. Additional research should explore broader domains that might be relevant among more ethnically and geographically diverse Black CaP survivors as regards accessing and utilising structured psychosocial support post-treatment.

## Data Availability

The data used in this systematic review are publicly available and included in the manuscript (see data extraction table and reference list).

## Appendix. Search strategies for the databases

### Medline all via OVID

1 exp Prostatic Neoplasms/ (125179)

2 (prostat* adj2 (cancer* or neoplasm* or tumor or tumour* or malign* or carcinoma* or metasta* or oncolog*)).ti,ab,kw. (130717)

3 (Cancer Survivors/ or (cancer* adj2 survivor*).ti,ab,kw.) and prostat*.ti,ab,kw. (1163)

4 1 or 2 or 3 [prostate cancer concept] (158977)

5 (black* or african* or caribbean or african-caribbean* or afro-caribbean* or african american* or (ethnic adj3 minorit*)).ti,ab,kw. (293407)

6 African Americans/ or African Continental Ancestry Group/ or Caribbean region/ (88720)

7 exp Africa/eh (7754)

8 exp Caribbean Region/eh (3457)

9 (BME or BAME).ti,ab,kw. (2197)

10 5 or 6 or 7 or 8 or 9 [black men] (325900)

11 4 and 10 [black men and prostate cancer] (3572)

12 (psychosocial or psychological or social or (support adj2 group) or emotional).ti,ab,kw. (797782)

13 support*.ti,ab,kw. (1490116)

14 Self-Help Groups/ (8991)

15 exp psychological phenomena/ or mental competency/ or mental health/ or mental processes/ or exp counseling/ or directive counseling/ or distance counseling/ or pastoral care/ or sex counseling/ (1804972)

16 exp social support/ or Community Networks/ (75545)

17 counsel*.ti,ab,kw. (105651)

18 exp peer group/ (20316)

19 peer*.ti,ab,kw. (87815)

20 exp Information Seeking Behavior/ (2243)

21 adaptation, psychological/ or exp attitude/ or exp health knowledge, attitudes, practice/ (632939)

22 Patient Education as Topic/ (83949)

23 exp religion/ (59916)

24 exp spirituality/ (7218)

25 exp Faith-Based Organizations/ (1707)

26 religio*.ti,ab,kw. (39213)

27 faith*.ti,ab,kw. (16114)

28 ((message or discussion) adj3 (board or internet or online)).ti,ab,kw. (1239)

29 (chatroom* or (chat adj room*)).ti,ab,kw. (355)

30 (Online adj3 forum).ti,ab,kw. (446)

31 (Social media or Facebook or Twitter or blog*).ti,ab,kw. (13866)

32 exp Consumer Health Information/ (8748)

33 blogging/ or social media/ (7701)

34 or/12-33 (4131946)

35 11 and 34 [3 concepts SG/OB] (906)

### Embase via OVID

#### Search strategy:

1. exp prostate cancer/ (211392)
2. (prostat* adj2 (cancer* or neoplasm* or tumor or tumour* or malign* or carcinoma* or metasta* or oncolog*)).ti,ab,kw. (198007)
3. (cancer Survivor/ or (cancer* adj2 survivor*).ti,ab,kw.) and prostat*.ti,ab,kw. (2121)
4. 1 or 2 or 3 (248100)
5. (black* or african* or caribbean or african-caribbean* or afro-caribbean* or african american* or (ethnic adj3 minorit*)).ti,ab,kw. (465185)
6. exp black person/ or african american/ or african brazilian/ or african caribbean/ (101313)
7. (BME or BAME).ti,ab,kw. (2168)
8. 5 or 6 or 7 (484101)
9. 4 and 8 (6274)
10. (psychosocial or psychological or social or (support adj2 group) or emotional).ti,ab,kw. (1047909)
11. support*.ti,ab,kw. (1921310)
12. self help/ (13337)
13. exp counseling/ (162741)
14. exp mental health/ (150033)
15. pastoral care/ (320)
16. social support/ (88485)
17. community care/ (54338)
18. counsel*.ti,ab,kw. (153719)
19. exp peer group/ (22854)
20. peer*.ti,ab,kw. (109526)
21. information seeking/ (3179)
22. coping behavior/ (56629)
23. attitude to health/ (109345)
24. patient education/ (111879)
25. exp religion/ (68674)
26. faith-based organization/ (139)
27. religio*.ti,ab,kw. (41632)
28. faith*.ti,ab,kw. (19363)
29. ((message or discussion) adj3 (board or internet or online)).ti,ab,kw. (1967)
30. (chatroom* or (chat adj room*)).ti,ab,kw. (484)
31. (Online adj3 forum).ti,ab,kw. (653)
32. (Social media or Facebook or Twitter or blog*).ti,ab,kw. (19832)
33. blogging/ (322)
34. social media/ (18187)
35. consumer health information/ (3767)
36. or/10-35 (3352544)
37. 9 and 36 (1285)

### APA PsycINFO via OVID

#### Search strategy:

1. exp Prostate/ and exp Neoplasms/ (1710)
2. (prostat* adj2 (cancer* or neoplasm* or tumor or tumour* or malign* or carcinoma* or metasta* or oncolog*)).ti,ab,id. (3041)
3. 1 or 2 (3079)
4. (black* or african* or caribbean or african-caribbean* or afro-caribbean* or african american* or (ethnic adj3 minorit*)).ti,ab,id. (131116)
5. exp blacks/ or exp minority groups/ (64335)
6. african cultural groups/ (2460)
7. (BME or BAME).ti,ab,id. (249)
8. 4 or 5 or 6 or 7 (142948)
9. 3 and 8 (442)
10. (psychosocial or psychological or social or (support adj2 group) or emotional).ti,ab,id. (1203016)
11. support*.ti,ab,id. (644814)
12. exp Group Counseling/ or exp Support Groups/ or exp Group Psychotherapy/ or exp Coping Behavior/ or exp Self-Help Techniques/ or exp Group Participation/ (92825)
13. exp Counseling/ (76578)
14. peers/ or peer counseling/ or peer relations/ (28053)
15. coping behavior/ or spiritual well being/ or “stress and coping measures”/ (47506)
16. exp Client Education/ (3921)
17. exp religion/ (70047)
18. exp spirituality/ (17404)
19. exp Faith Based Organizations/ (313)
20. (religio* or faith*).ti,ab,id. (83649)
21. ((message or discussion) adj3 (board or internet or online)).ti,ab,id. (1951)
22. (chatroom* or (chat adj room*)).ti,ab,id. (703)
23. (Online adj3 forum).ti,ab,id. (611)
24. (Social media or Facebook or Twitter or blog*).ti,ab,id. (16960)
25. exp Social Media/ (13310)
26. exp Online Social Networks/ or exp Blog/ (7809)
27. or/10-26 (1779294)
28. 9 and 27 (188)

### CINAHL (ran: 17.01.2020)

1. (MH “Prostatic Neoplasms+”)

2. (prostat* N2 (cancer* OR neoplasm* OR tumor* OR tumour* OR malign* OR carcinoma* OR metasta* OR oncolog*))

3. ((MH “Cancer Survivors”) OR (cancer* N2 survivor*)) AND prostat*

4. 1 OR 2 OR 3

5. (black* OR African* OR Caribbean OR African-Caribbean* OR Afro-Caribbean* OR African American* OR (ethnic N3 minorit*))

6. (MH “Blacks”) OR (MH “African Continental Ancestry Group”) OR (MH “West Indies+/EH”)

7. (MH “Africa+/EH”)

8. *Searched for this in line 6 instead*

9. (BME or BAME)

10. 5 OR 6 OR 7 OR 9

11. 4 AND 10

12. (psychosocial OR psychological OR social OR (support N2 group) OR emotion*)

13. support*

14. (MH “Support Groups”)

15. (MH “Psychological Processes and Principles+”) OR (*No equivalent MH to mental competency*) OR (MH “Mental Health”) OR (MH “Mental Processes”) OR (MH “Counseling+”) OR (*No equivalent MH to directive counselling*) OR (*No equivalent MH to distance counselling*) OR (MH “Spiritual Care”) OR (MH “Sexual Counseling”)

16. (MH “Support, Psychosocial+”) OR (MH “Community Networks”)

17. Counsel*

18. (MH “Peer Group”) *Unable to explode*

19. peer*

20. (MH “Information Seeking Behavior”) *Unable to explode*

21. (MH “Adaptation, Psychological”) OR (MH “Attitude+”) OR (MH “Health Knowledge”)

22. (MH “Patient Education”)

23. (MH “Religion and Religions+”)

24. (MH “Spirituality”) *Unable to explode*

25. (MH “Faith-Based Organizations”) *Unable to explode*

26. religio*

27. faith*

28. ((message OR discussion) N3 (board OR internet OR online))

29. (chatroom* OR (chat N1 room*))

30. (online N3 forum)

31. (Social media OR Facebook OR Twitter OR Blog*)

32. (MH “Consumer Health Information+”)

33. (MH “Blogs”) OR (MH “Social Media+”)

34. or/12-13

35. 11 AND 34

CINAHL — 553 results

### WoS (ran 26.01.2020)

1. (prostat* NEAR/1 (cancer* OR neoplasm* OR tumor* OR tumour* OR malign* OR carcinoma* OR metasta* OR oncolog*))

2. ((cancer* NEAR/2 survivor*) AND prostat*)

3. 1 OR 2

4. (black* OR African* OR Caribbean OR African-Caribbean* OR Afro-Caribbean* OR African American* OR (ethnic NEAR/3 minorit*))

5. (BME OR BAME)

6. 4 OR 5

7. 3 AND 6

8. (psychosocial OR psychological OR social OR (support NEAR/2 group) OR emotion*)

9. support*

10. counsel*

11. peer*

12. religio*

13. faith*

14. ((message OR discussion) NEAR/3 (board OR internet OR online))

15. (chatroom* OR (chat NEAR/1 room*))

16. (online NEAR/3 forum)

17. (Social media OR Facebook OR Twitter OR Blog*)

18. or 8-17

19. 7 AND 18

Results — 803 (687 articles only)

### Cochrane (ran 07.02.2020)

1. MeSH descriptor: [Prostatic Neoplasms] Explode all trees

2. (prostat* NEAR/2 (cancer* OR neoplasm* OR tumor* OR tumour* OR malign* OR carcinoma* OR metasta* OR oncolog*))

3. MeSH descriptor: [Cancer Survivors] this term only OR (cancer* NEAR/2 survivor*) AND prostat*

4. 1 OR 2 OR 3

5. (black* OR African* OR Caribbean OR African-Caribbean* OR Afro-Caribbean* OR African American* OR (ethnic NEAR/3 minorit*))

6. MeSH descriptor: [African Continental Ancestry Group] this term only OR MeSH descriptor: [Caribbean Region] explode all trees

7. MeSH descriptor: [Africa] explode all trees

8. BME OR BAME

9. 5 OR 6 OR 7 OR 8

10. 4 AND 9

11. (psychosocial OR psychological OR social OR (support NEAR/2 group) OR emotion*)

12. support*

13. MeSH descriptor: [Self-Help Groups] this term only

14. MeSH descriptor: [Psychological Phenomena] explode all trees OR MeSH descriptor: [Mental Competency] explode all trees OR MeSH descriptor: [Mental Health] this term only OR MeSH descriptor: [Mental Health] this term only OR MeSH descriptor: [Counseling] explode all trees OR MeSH descriptor: [Directive Counseling] this term only OR MeSH descriptor: [Distance Counseling] this term only OR MeSH descriptor: [Spirituality] this term only OR MeSH descriptor: [Sex Counseling] this term only

15. MeSH descriptor: [Psychosocial Support Systems] explode all trees OR MeSH descriptor: [Community Networks] this term only

16. Counsel*

17. MeSH descriptor: [Peer Group] explode all trees

18. Peer*

19. MeSH descriptor: [Information Seeking Behavior] explode all trees

20. MeSH descriptor: [Adaptation, Psychological] this term only OR MeSH descriptor: [Attitude] explode all trees OR MeSH descriptor: [Health Knowledge, Attitudes, Practice] this term only

21. MeSH descriptor: [Patient Education as Topic] this term only

22. MeSH descriptor: [Religion] explode all trees

23. MeSH descriptor: [Spiritual Therapies] this term only

24. MeSH descriptor: [Faith-Based Organizations] explode all trees

25. religio*

26. faith*

27. ((message OR discussion) NEAR/3 (board OR internet OR online))

28. (chatroom* OR (chat NEAR/1 room*))

29. (online NEAR/3 forum)

30. (Social media OR Facebook OR Twitter OR Blog*)

31. MeSH descriptor: [Consumer Health Information] explode all trees

32. MeSH descriptor: [Consumer Health Information] explode all trees OR MeSH descriptor: [Social Media] explode all trees

33. 11-32

34. 10 AND 33

Results — 232 (48 reviews, 176 trials, 8 protocols)

### Scopus

( ( TITLE-ABS-KEY ( ( *prostat** W/2 ( *cancer** OR *neoplasm** OR *tumor* OR *tumour** OR *malign** OR *carcinoma** OR *metasta** OR *oncolog** ) ) ) ) AND ( TITLE-ABS-KEY ( *black** OR *african** OR *caribbean* OR *african-caribbean** OR *afro-caribbean** OR *bme* OR *bame* OR *african* AND *american** OR ( *ethnic* W/3 *minorit** ) ) ) ) AND ( ( TITLE-ABS-KEY ( *psychosocial* OR *psychological* OR *social* ) OR TITLE-ABS-KEY ( ( *support* W/2 *group* ) OR *emotional** OR *support** OR *counsel** OR *peer** OR *religio** OR *faith** ) OR TITLE-ABS-KEY ( ( ( *message* OR *discussion* OR *online* ) W/2 ( *board* OR *online* OR *internet* OR *forum** ) ) OR *social* AND *media* OR *blog** OR *facebook* OR *twitter* ) ) ) AND NOT INDEX ( *medline* )
